# Evolution of Doppler ultrasound in obstetric imaging: a slow(flow) step forward

**DOI:** 10.3389/fmed.2026.1878231

**Published:** 2026-07-17

**Authors:** Simcha Yagel, Sarah M. Cohen, Tomer Shwartz, Dan V. Valsky

**Affiliations:** Division of Obstetrics and Gynecology, Hadassah University Medical Center, Jerusalem, Israel

**Keywords:** Doppler, fetal venous system, placenta accreta spectrum, prenatal diagnosis, Slow*flow*HD

## Abstract

Early applications of fetal Doppler evaluation drew on parameters derived from adult vascular examination, primarily applied to identifying fetuses at risk for growth restriction and hypoxia. The integration of color Doppler into real-time imaging facilitated a shift toward structural-functional correlation, enabling clinicians to visualize fetal cardiac outflow tracts, valve regurgitant jets, and the complex umbilical–venous connections alongside standard grayscale anatomy. Power Doppler extended these capabilities to slow flow and small caliber vascular structures. Doppler modalities have been crucial in delineating the fetal cardiovascular system, increasingly detecting complex congenital heart disease (CHD) prenatally, and illuminating cardiac and non-cardiac vascular structures that previously posed a challenge to visualization, including cerebral, hepatic, and placental capillary beds. Highly sensitive Doppler technologies such as Slow*flow*HD as well as MV-Flow and LumiFlow, exemplify the convergence of anatomical and physiological imaging, such as fetal venous sinuses and deep cerebral venous return, to allow brain perfusion assessment at earlier gestational stages. Although Slow*flow*HD has been shown to have added value for diagnosis, it seems to be an underused modality. Here we show several examples drawn from our experience with Slow*flow*HD, in both routine scanning and anomalous cases spanning various organ systems and stages of gestation. While there is a learning curve in optimizing image acquisition and interpretation of microvascular anatomy, it should not deter practitioners proficient in Doppler techniques. We recommend incorporation of Slow*flow*HD and comparable microvascular imaging technologies into the sonographic armamentarium.

## From hemodynamic monitoring to anatomical discovery

Early applications of fetal Doppler evaluation drew on parameters derived from adult vascular examination. Early practitioners applied the continuous-wave Doppler, devising the Pulsatility Index for fetal focused hemodynamic assessment, especially the umbilical and uteroplacental circulations (1, 2), identifying fetuses at risk for growth restriction and hypoxia (3, 4). Pulsed-wave Doppler allowed for site-specific sampling and enabled clinicians to evaluate flow velocity waveforms and indices (5, 6), forming the basis of prenatal cardiovascular surveillance. The integration of color Doppler into real-time imaging facilitated a shift toward structural-functional correlation, allowing clinicians to visualize fetal cardiac outflow tracts, valve regurgitant jets, and the complex umbilical–venous connections alongside standard grayscale anatomy (7–9).

By the mid-1990s, power Doppler, which measures the amplitude rather than frequency of returning echoes, extended these capabilities to slow flow and small caliber vascular structures (10, 11). This provided greater insight into organ-specific vasculature such as hepatic, renal, and cerebral microcirculation (12–17). Studies integrating power Doppler with 3D image reconstruction improved visualization of fetal organ development and placental vascular morphology, enabling functional correlations between perfusion and morphogenesis (18, 19).

## Expanding visualization of the fetal cardiovascular system

Doppler modalities have been crucial in delineating the fetal cardiovascular system, improving prenatal detection of complex congenital heart disease (CHD). Color or bidirectional power Doppler and analysis with Fetal Intelligent Navigation Echocardiography (FINE) allows generation of standard fetal echocardiography views with Doppler information and improves visualization of abnormal cardiac anatomy and flow patterns in fetuses with congenital heart disease (20). Techniques like Spatiotemporal Image Correlation (STIC) and directional power Doppler allowed dynamic flow visualization through the cardiac cycle, creating virtual 3D renderings that deepened anatomical comprehension and diagnostic precision (18, 21, 22).

As sensitivity improved, Doppler imaging illuminated cardiac and non-cardiac vascular structures that previously posed a challenge to visualization, including cerebral, hepatic, and placental capillary beds (2, 11, 12, 14–17, 23, 24), by employing adaptive clutter suppression and low pulse repetition frequencies to reveal perfusion patterns.

## The contribution of Slow*flow*HD and other microvascular imaging modalities

Highly sensitive Doppler technologies such as Slow*flow*HD (25–32), as well as MV-Flow and LumiFlow (23, 24, 33–38), exemplify the convergence of anatomical and physiological imaging. Capable of detecting flow velocities below 1 cm/s, these modalities visualize minute vessels, thereby offering a new physiological dimension to obstetric imaging.

For example, this technology has enabled visualization of fetal venous sinuses and deep cerebral venous return to allow brain perfusion assessment at earlier gestational stage than conventional Doppler (25, 28, 35, 38, 39). A 2025 study demonstrated how Slow*flow*HD can map the placental villous tree (27), differentiating structural vascular archetypes and revealing abnormal arborization patterns in fetuses with congenital heart disease, directly linking microanatomical imaging to clinical pathophysiology (27).

## Impact on imaging fetal anatomy

This technological progression, from M-mode to early Doppler velocimetry and color mapping, to Slow*flow*HD, has shifted fetal ultrasound from simple flow detection to comprehensive angiographic and anatomical mapping at the microvascular level. Modern Doppler systems now reveal the vascular architecture of fetal organs and quantify perfusion in embryonic and fetal tissues across all gestational stages.

Although Slow*flow*HD has been shown to be safe (31) and effective (25–30, 32, 39), and capable of imaging fetal vasculature not otherwise available for analysis (28), and has been shown to have added value for diagnosis, it seems to be an underused modality. While Slow*flow*HD does have the disadvantage of being non-directional and therefore does not allow for measurement of standard Doppler indices, it has advantages, particularly in visualizing subtle structures at very early gestational ages. Slow*flow*HD does require a learning curve to master the power and balance controls to optimize image acquisition.

In our center, we applied Slow*flow*HD to a convenience sample of 157 fetuses presenting for fetal anatomy scanning in the late first or early second trimester. This comprised a subset of a non-inferiority study comparing alternative timing protocols of the nuchal translucency and anatomy scans (40). Briefly, during the Doppler portion of the scan, the Slow*flow*HD modality was applied via dedicated control in the user interface. The operator then adjusted the power and balance settings to ensure that the color appropriately demonstrated the target vessels. Examinations were performed in accordance with the ALARA principle (41): the thermal and mechanical indices for were TIs: 0.2; Tib: 0.2; and MI: 0.8, respectively, well below the recommended ranges. Dwell time was minimized as far as possible. The additional scanning time to acquire the target anatomy was approximately 90 s.

We showed that following a learning curve, the target vessels were consistently imaged (28) (Table 1). Slow*flow*HD facilitated the visualization of subtle vascular structures that are not amenable to visualization with high definition color Doppler (28) (HD-Doppler). Our observations are based on our experience with Slow*flow*HD, other proprietary technologies can produce similar results, as reported in the literature (23, 24, 33–38).

**Table 1:** Comparison of visualization rates of fetal vessels during early exams, using Slow*flow*HD vs. HD-Doppler.

Structures studied	Slow*flow*HD (*N* = 157) *n* (%)	HD Doppler (*N* = 157) *n* (%)	Difference in % [95% CI]	*p*-value^a^
Cerebral arteries				
Anterior cerebral	153 (97.5)	71 (45.2)	52.2 [43.3, 61.2]	*p* < 0.001
Pericallosal	157 (100)	58 (36.9)	63.1 [55.0, 71.1]	*p* < 0.001
Cerebral venous structures				
Superior sagittal sinus	157 (100)	100 (63.7)	36.3 [28.4, 44.2]	*p* < 0.001
Straight sinus	148 (94.3)	76 (48.4)	45.9 [36.4, 55.3]	*p* < 0.001
Vein of Galen	146 (93.0)	21 (13.4)	79.6 [71.8, 87.4]	*p* < 0.001
Torcular Herophili	101 (64.3)	24 (15.3)	49.0 [39.3, 58.8]	*p* < 0.001
Thoracic arteries				
Subclavian	128 (81.5)	33 (21.0)	60.5 [51.1, 69.9]	*p* < 0.001
Internal thoracic	128 (81.5)	17 (10.8)	70.7 [62.4, 79.0]	*p* < 0.001
Aortic arch bifurcations	98 (62.4)	34 (21.6)	40.8 [30.6, 50.9]	*p* < 0.001
Thoracic veins				
Pulmonary veins (2)	157 (100)	133 (84.7)	15.3 [9.6, 21.0]	*p* < 0.001
Azygos	152 (96.8)	70 (44.6)	52.2 [43.3, 61.1]	*p* < 0.001
Abdominal veins				
Portal system	157 (100)	71 (45.2)	54.8 [46.6, 62.9]	*p* < 0.001
Hepatic system	157 (100)	78 (49.7)	50.3 [42.1, 58.5]	*p* < 0.001
IVC	157 (100)	154 (98.1)	1.9 [−0.2, 4.0]	*p* = 0.25
Umbilical	157 (100)	157 (100)	0.0 [0.0, 0.0]	*p* = 1.00
Left portal and DV	157 (100)	139 (88.5)	11.5 [6.4, 16.5]	*p* < 0.001
Cardiac				
Four chamber view	157 (100)	157 (100)	0.0 [0.0, 0.0]	*p* = 1.00
RVOT	157 (100)	157 (100)	0.0 [0.0, 0.0]	*p* = 1.00
LVOT	157 (100)	157 (100)	0.0 [0.0, 0.0]	*p* = 1.00
3VT	157 (100)	157 (100)	0.0 [0.0, 0.0]	*p* = 1.00

a Chi-square test.

Here we show several examples drawn from our experience in both routine scanning and anomalous cases, spanning various organ systems and stages of gestation. We have limited our examples to fetal-maternal scanning to maintain clarity and focus, however Slow*flow*HD is valuable in gynecological applications as well. Care must always be taken to adjust the power and balance functions as necessary to optimize visualization. These examples illustrate capabilities but are not intended to demonstrate superiority in detection rates of anomalies as compared to other modalities, which was beyond the purpose of this Perspective (Supplementary Table 1 summarizes the details of the figures).

**Figure 1:**
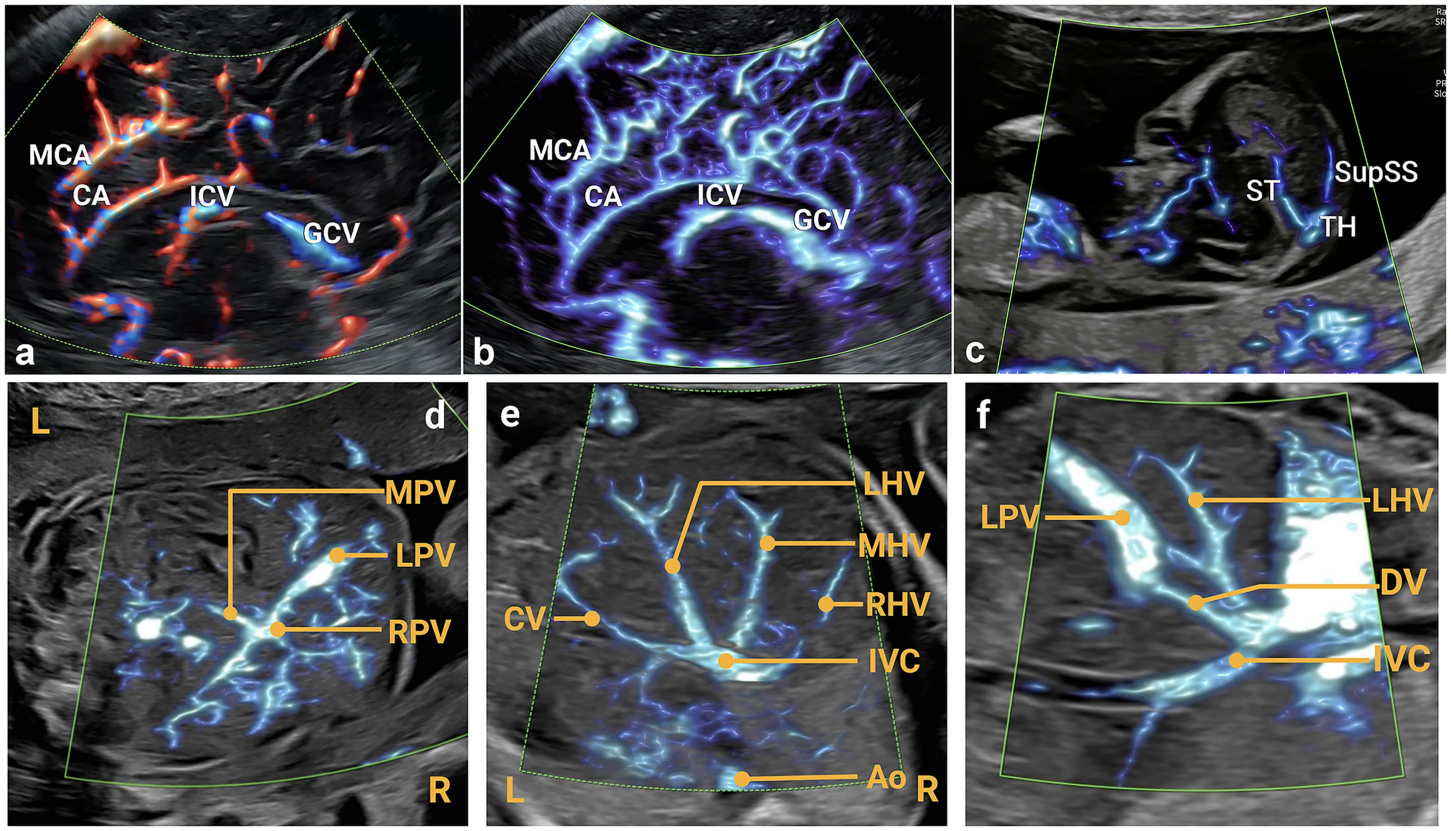
**(a–c)** Compare brain vasculature imaged with HD-Doppler vs. Slow*flow*HD. In this normal fetus imaged at 34 weeks GA, note the marked difference in the quantity and scale of the vessels imaged and their clarity, comparing HD-Doppler **(a)** and Slow*flow*HD **(b)**. In early gestation, vasculature not usually amenable to imaging is seen in detail. In this 13-weeks fetus, the straight sinus (ST), superior sagittal sinus (SupSS), and even the Torcular Herophili (TH) are imaged **(c)**. (MCA, mid-cerebral artery; CA, callosal artery; ICV, internal cerebral vein; GCV, great cerebral vein). **(d–f)** The three planes of the precordial venous system imaged in Slow*flow*HD. **(a)** The lateral transverse plane through the fetal abdomen from left to right. (LPV; left portal vein; MPV, main portal vein; RPV, right portal vein. L and R indicate left and right). **(b)** Anterior–posterior transverse plane through the upper fetal abdomen showing the normal hepatic veins, and to the left of the LHV, the caudate lobe vein. (Ao, aorta; CV, caudate vein; LHV, left hepatic vein; MHV, middle hepatic vein; RHV, right hepatic vein. L and R indicate left and right). **(c)** Typical longitudinal plane showing the left portal vein (LPV); left hepatic vein (LHV); ductus venosus (DV); and inferior vena cava (IVC).

Figures 1a,b demonstrates the brain vasculature visualized in a 34-week fetus in HD-Doppler vs. Slow*flow*HD, while in a 13-week fetus (Figure 1c) vascular structures as subtle as the Torcular Herophili are imaged.

We have shown that imaging the precordial venous system is accessible, robust, and informative in routine fetal anatomy scanning from 13 weeks’ gestation (40, 42, 43). Figures 1d–f shows the three planes of the precordial venous system (43) scanned with Slow*flow*HD. Application of the Slow*flow*HD allows visualization of the caudate lobe vein in addition to the three hepatic veins.

Supplementary figures demonstrate Slow*flow*HD in other organ systems. Imaging the corpus callosum to confirm or exclude a partial agenesis is always challenging. The fetus depicted in Supplementary Figure 1 was scanned at 23 weeks. HD-Doppler showed the interrupted callosal artery, while Slow*flow*HD provided more detailed visualization of the surrounding vasculature, including false continuation of the callosal artery (Supplementary Figure 1b), a potential limitation.

In another fetus with suspected brain lesion (Supplementary Figure 2), the grayscale image showed an area of echogenicity, first suspected to be a hemorrhage in the left hemisphere, possibly carrying an unfavorable outcome. Fetal MRI suggested a differential diagnosis of sub-pial hemorrhage or capillary telangiectasia. Slow*flow*HD in this case surpassed fetal MRI as well as Doppler ultrasound, demonstrating small vessels within the finding that supported the diagnosis of telangiectasia. After delivery, diagnosis of benign telangiectasia was confirmed.

Supplementary Figure 3a shows normal pulmonary veins imaged with Slow*flow*HD in a 13-week fetus. With particular attention to power and balance settings, fetal cardiac imaging, with its higher flow rates, is also amenable to Slow*flow*HD scanning (32). Supplementary Figure 3b depicts the vessels of the fetal mediastinum with the typical thy-box sign (44–46) as well as smaller vessels not routinely visualized in this plane.

Coarctation of the aorta presents with a spectrum of findings that can evolve during gestation. Two cases diagnosed in our center exemplify this (Supplementary Figure 4). In one fetus, the 3VT plane appeared normal on HD-Doppler however, Slow*flow*HD of the aortic isthmus showed the severely narrowed aorta, with *Z*-score −4.81 (Supplementary Figures 4a,b). In the second case, the fetus presented with a critically narrow aortic arch and dilated pulmonary artery (PA), consistent with tubular aortic coarctation. Supplementary Figure 4c shows the HD-Doppler scan of the dilated PA while Supplementary Figure 4d depicts the dilated PA as well as the narrow aortic arch and characteristic bifurcations.

In a routine early fetal anatomy scan at 13 weeks, a dilated aortic arch was observed in the 3VT plane, raising suspicion of tetralogy of Fallot (Supplementary Figure 5a); other planes were consistent with the diagnosis. However, only Slow*flow*HD successfully depicted the severely stenotic main pulmonary artery (Supplementary Figure 5b).

We have also found Slow*flow*HD to be valuable in maternal scanning to depict subtle vascular structures. A woman with history of cesarean delivery presented at 14 weeks for evaluation of suspected cesarean scar niche. The characteristic niche was readily visualized in grayscale (Supplementary Figure 6a). HD-Doppler (Supplementary Figure 6b) raised suspicion for placenta accreta but did not show bladder invasion. Early diagnosis of placenta percreta was made possible by the application of Slow*flow*HD, which confirmed invasion to the maternal bladder (Supplementary Figure 6c).

## Limitations

Slow*flow*HD is a valuable modality however, it is not without its limitations. Slow*flow*HD and similar tools (MV-Flow, LumiFlow) are available on specific advanced ultrasound systems. The cases we present here are all from a single referral center with a high volume of fetal anomalies and extensive expertise, particularly in the diagnosis of fetal vascular anomalies. Our approach presupposes a familiarity with Doppler scanning; incorporation of Slow*flow*HD into routine scanning will require additional examination time however, following a learning curve we found Slow*flow*HD to be accessible and intuitive in its application, and the added time required to be minimal. Our earlier study design did not include inter- or intra-observer or cross-equipment comparisons: these are potential avenues of future research.

## Conclusion

Slow*flow*HD may provide added value in routine obstetric scanning and in assessment of both fetal and maternal anomalies. However, this and other microvascular imaging modalities appear to be underutilized in clinical practice. Although there is a learning curve in optimizing image acquisition and interpretation of microvascular anatomy, it should not deter practitioners proficient in Doppler techniques. We recommend incorporation of Slow*flow*HD and comparable microvascular imaging technologies into the sonographic armamentarium.

## Data Availability

The raw data supporting the conclusions of this article will be made available by the authors, without undue reservation.

## Supplementary material

SUPPLEMENTARY FIGURE 1 Partial agenesis of the corpus callosum. Compare the HD-Doppler scan **(a)** of this case of partial agenesis of the corpus callosum imaged at 23 weeks, and the more extensive vascular structure visualized with Slow*flow*HD. However, note the false continuation of the callosal artery **(b)**.

SUPPLEMENTARY FIGURE 2 Cerebellar hyperechogenicity at 22 weeks GA. The finding was originally diagnosed as an infratentorial vascular malformation, shown here in grayscale **(a)**. Fetal MRI proposed a differential diagnosis of sub-pial hemorrhage or capillary telangiectasia. Slow*flow*HD demonstrated small vessels within the finding **(b)** supporting the diagnosis of telangiectasia **(c)**, which was confirmed following uneventful delivery.

SUPPLEMENTARY FIGURE 3 First trimester imaging of the pulmonary veins and the thy-box plane **(a)**. Normal pulmonary veins are demonstrated with Slow*flow*HD in this 13-week fetus **(b)**.

SUPPLEMENTARY FIGURE 4 Coarctation of the aorta. Two cases of aortic coarctation imaged in Slow*flow*HD, compared with HD-Doppler image. The 3VT plane appears relatively normal **(a)**, however Slow*flow*HD showed the narrow aortic isthmus **(b)**. In another case, HD-Doppler showed only the dilated pulmonary artery (PA) **(c)**, while Slow*flow*HD demonstrated the critically narrow aortic arch (AoA) and dilated PA in a case of tubular aortic coarctation **(d)**.

SUPPLEMENTARY FIGURE 5 Tetralogy of Fallot. Dilated aortic arch is seen in the 3VT plane in a case of tetralogy of Fallot at 13 weeks **(a)**. Only Slow*flow*HD depicted the severely stenotic main pulmonary artery (MPA) **(b)**. (AoA, aortic arch; LPA, left pulmonary artery; RPA, right pulmonary artery).

SUPPLEMENTARY FIGURE 6 Placenta percreta. Early diagnosis of placenta percreta at 14 weeks was made possible by the application of Slow*flow*HD. While the characteristic niche is visible in grayscale **(a)**, the Doppler image **(b)** was suspicious for placenta accreta but did not show bladder invasion. Slow*flow*HD showed invasion to the maternal bladder **(c)**.
