# Supplementary figures and images for: Evolution of Doppler ultrasound in obstetric imaging: a slow(flow) step forward
Source: Front Med (Lausanne). 2026 Jul 17;13:1878231. doi: 10.3389/fmed.2026.1878231 (PMC13423865; doi:10.3389/fmed.2026.1878231)

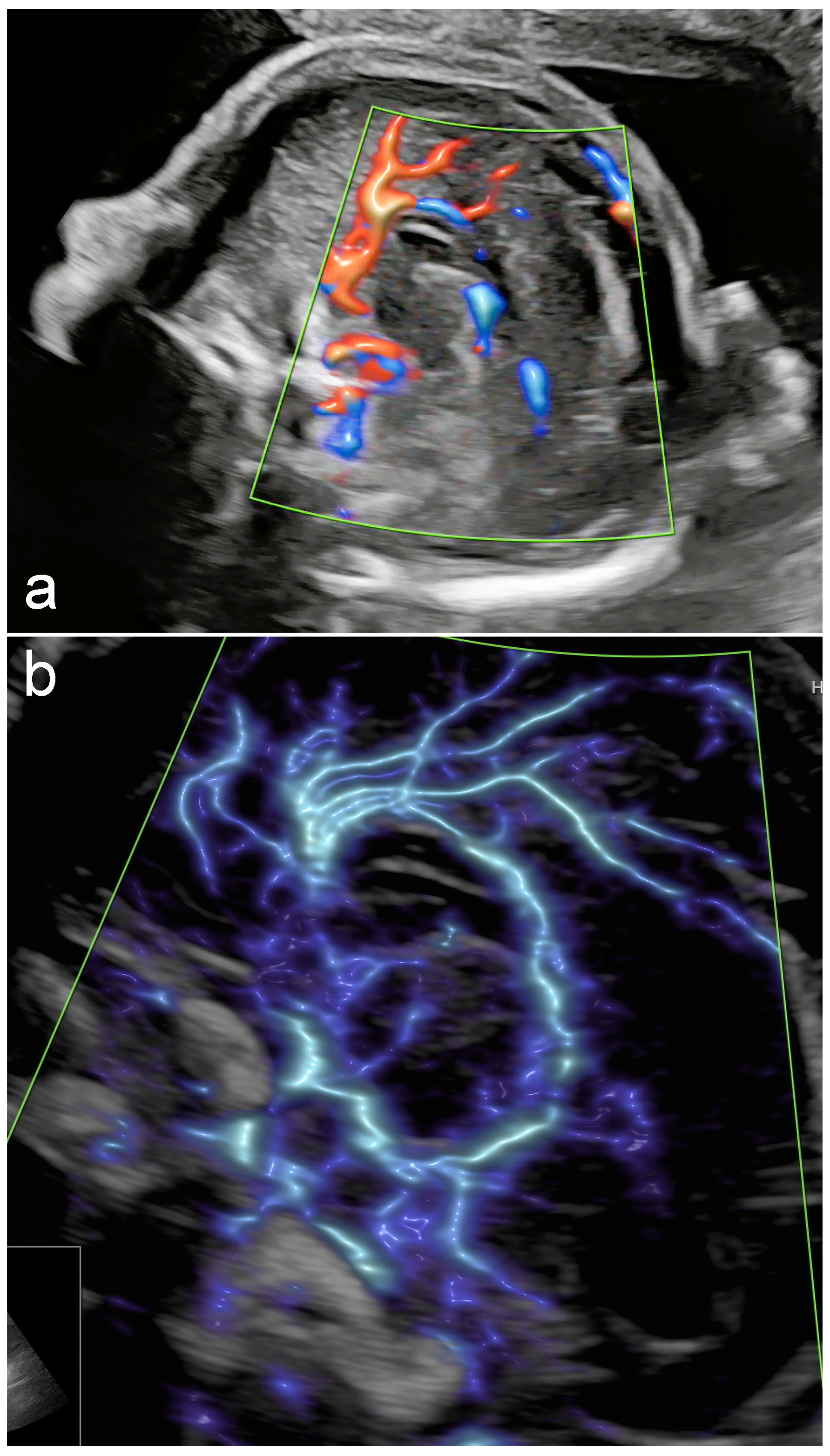

Supplement: SUPPLEMENTARY FIGURE 1 — Partial agenesis of the corpus callosum. Compare the HD-Doppler scan (a) of this case of partial agenesis of the corpus callosum imaged at 23 weeks, and the more extensive vascular structure visualized with SlowflowHD. However, note the false continuation of the callosal artery (b). [file Image_1.tif]

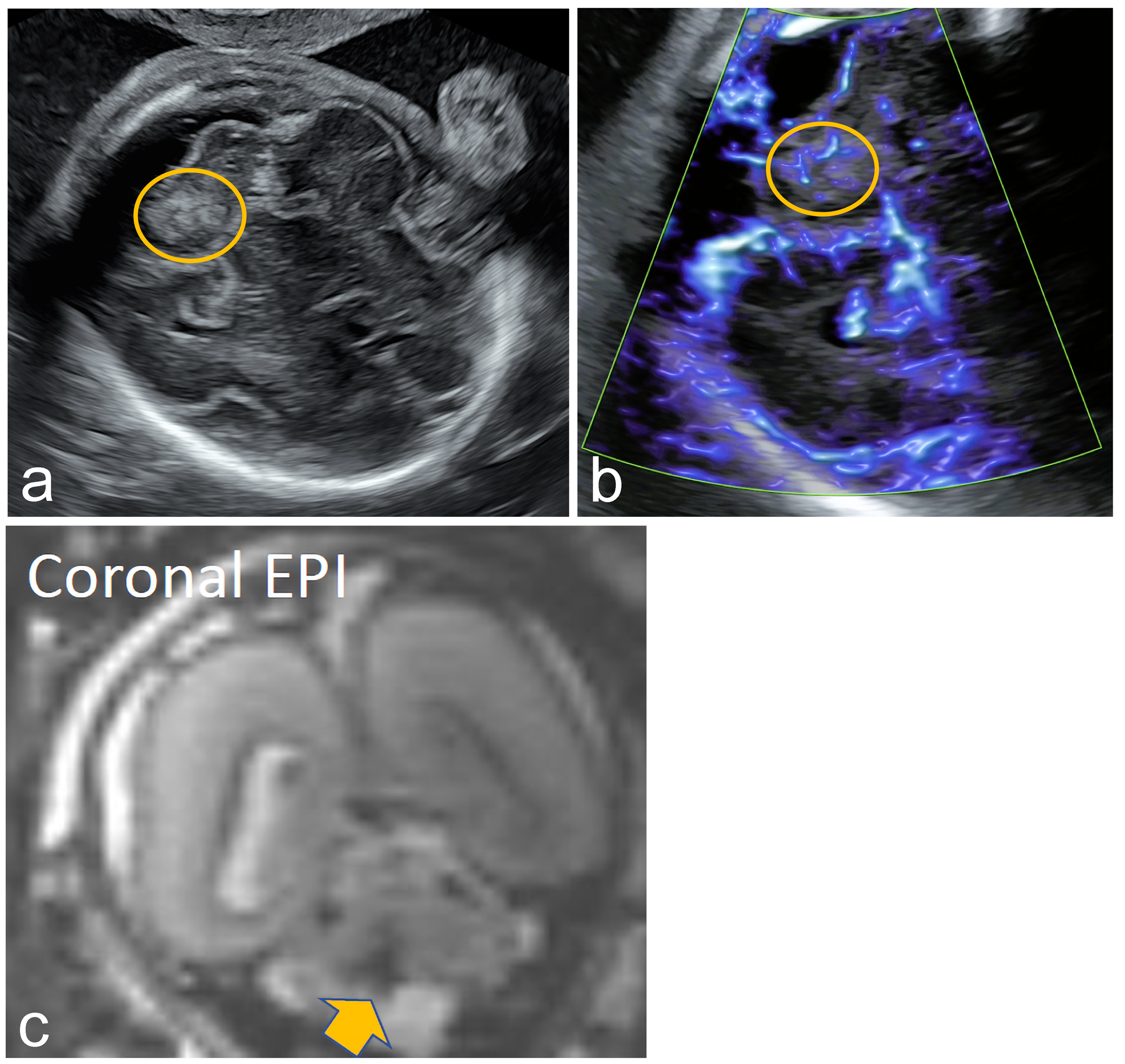

Supplement: SUPPLEMENTARY FIGURE 2 — Cerebellar hyperechogenicity at 22 weeks GA. The finding was originally diagnosed as an infratentorial vascular malformation, shown here in grayscale (a). Fetal MRI proposed a differential diagnosis of sub-pial hemorrhage or capillary telangiectasia. SlowflowHD demonstrated small vessels within the finding (b) supporting the diagnosis of telangiectasia (c), which was confirmed following uneventful delivery. [file Image_2.tif]

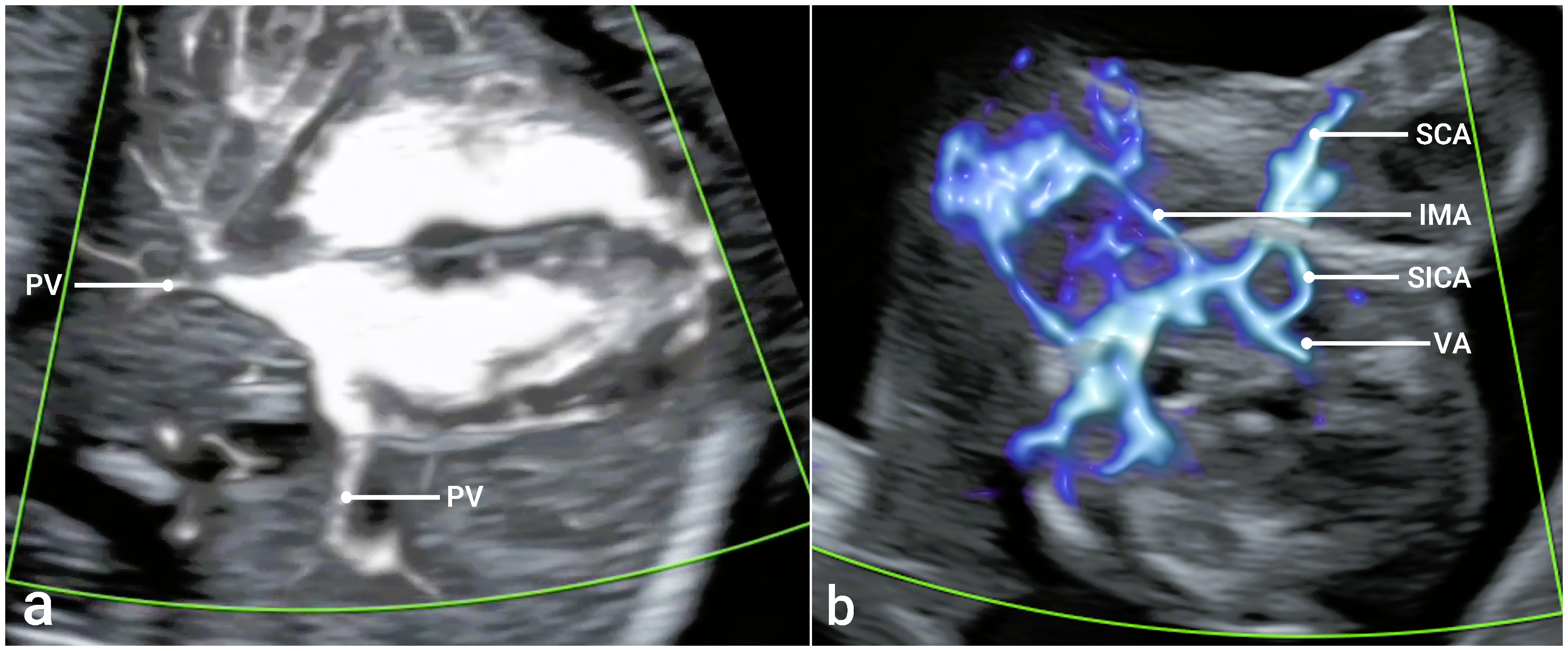

Supplement: SUPPLEMENTARY FIGURE 3 — First trimester imaging of the pulmonary veins and the thy-box plane (a). Normal pulmonary veins are demonstrated with SlowflowHD in this 13-week fetus (b). [file Image_3.tif]

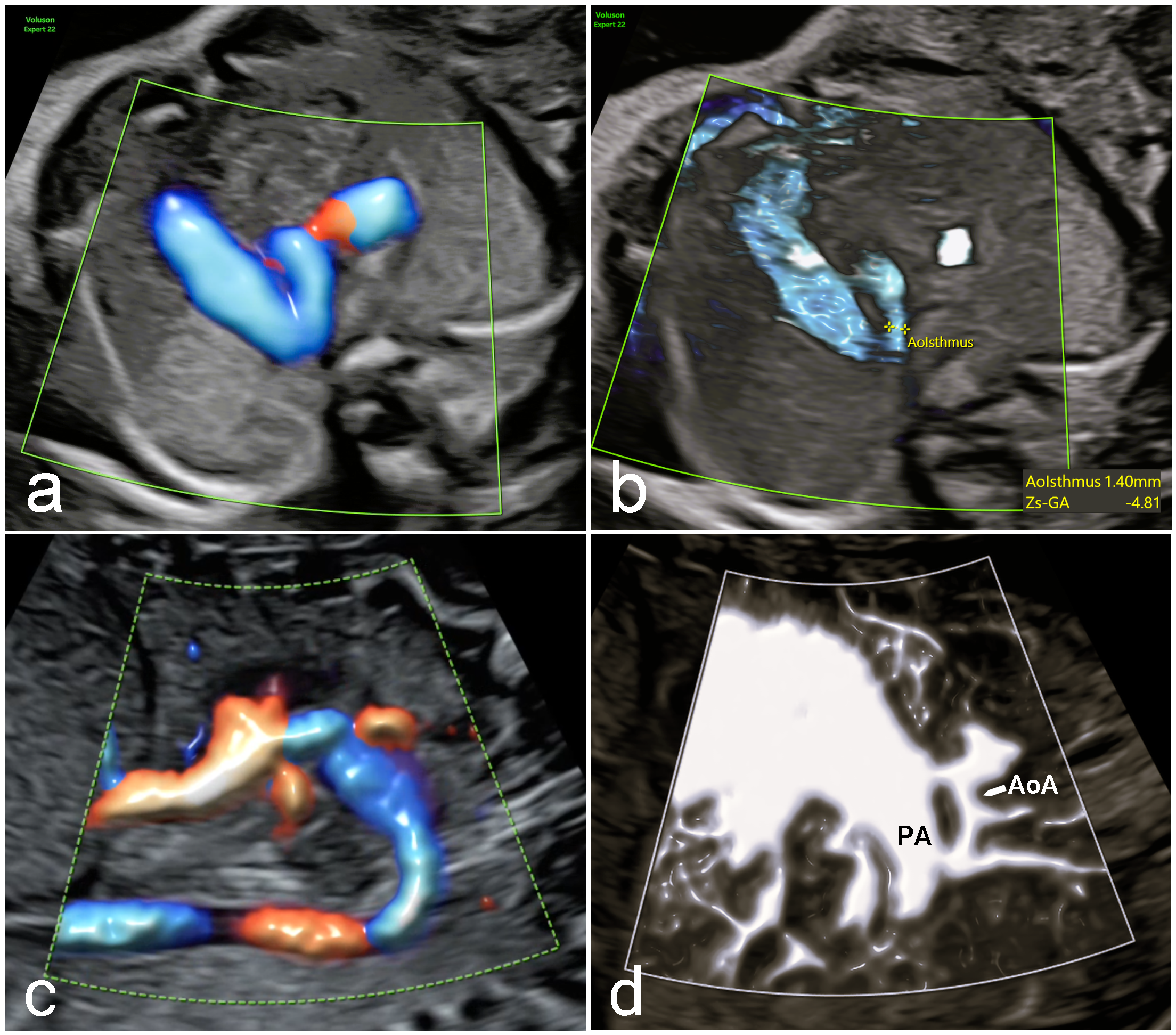

Supplement: SUPPLEMENTARY FIGURE 4 — Coarctation of the aorta. Two cases of aortic coarctation imaged in SlowflowHD, compared with HD-Doppler image. The 3VT plane appears relatively normal (a), however SlowflowHD showed the narrow aortic isthmus (b). In another case, HD-Doppler showed only the dilated pulmonary artery (PA) (c), while SlowflowHD demonstrated the critically narrow aortic arch (AoA) and dilated PA in a case of tubular aortic coarctation (d). [file Image_4.tif]

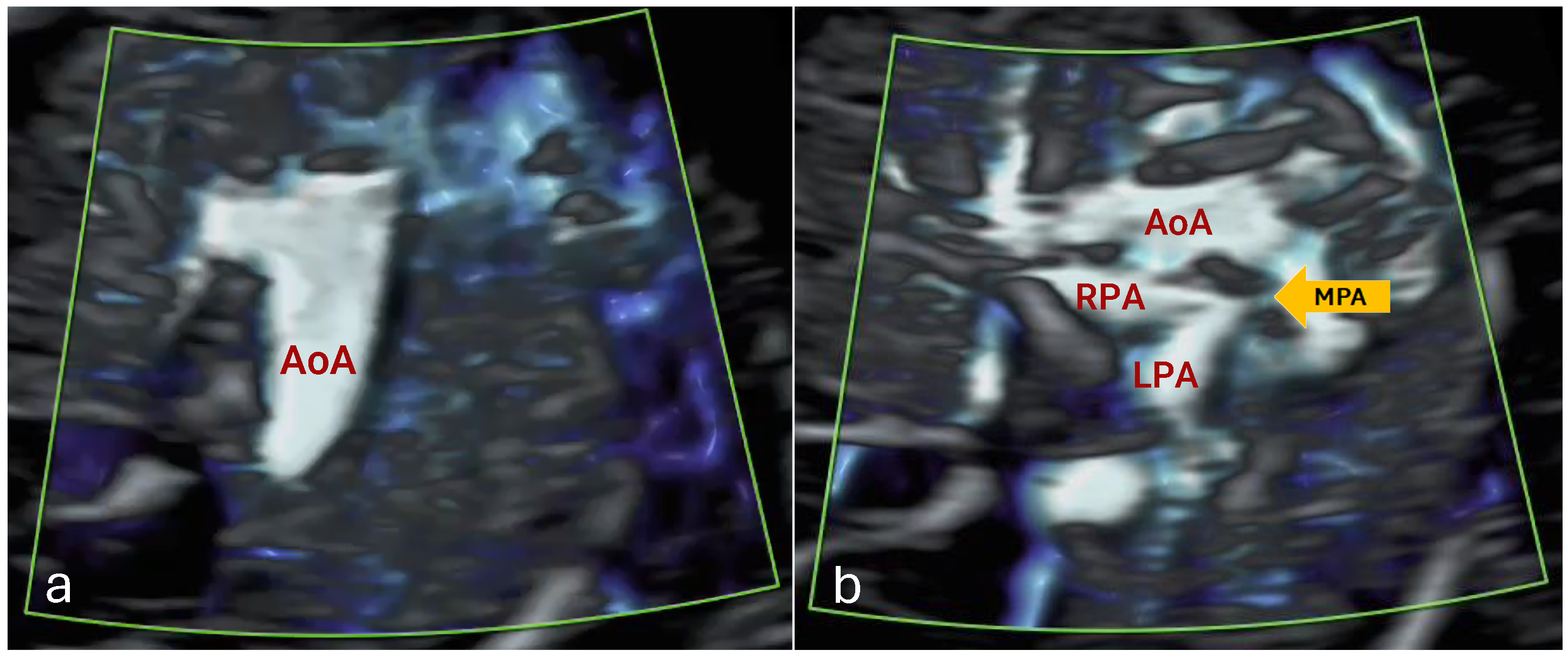

Supplement: SUPPLEMENTARY FIGURE 5 — Tetralogy of Fallot. Dilated aortic arch is seen in the 3VT plane in a case of tetralogy of Fallot at 13 weeks (a). Only SlowflowHD depicted the severely stenotic main pulmonary artery (MPA) (b). (AoA, aortic arch; LPA, left pulmonary artery; RPA, right pulmonary artery). [file Image_5.tif]

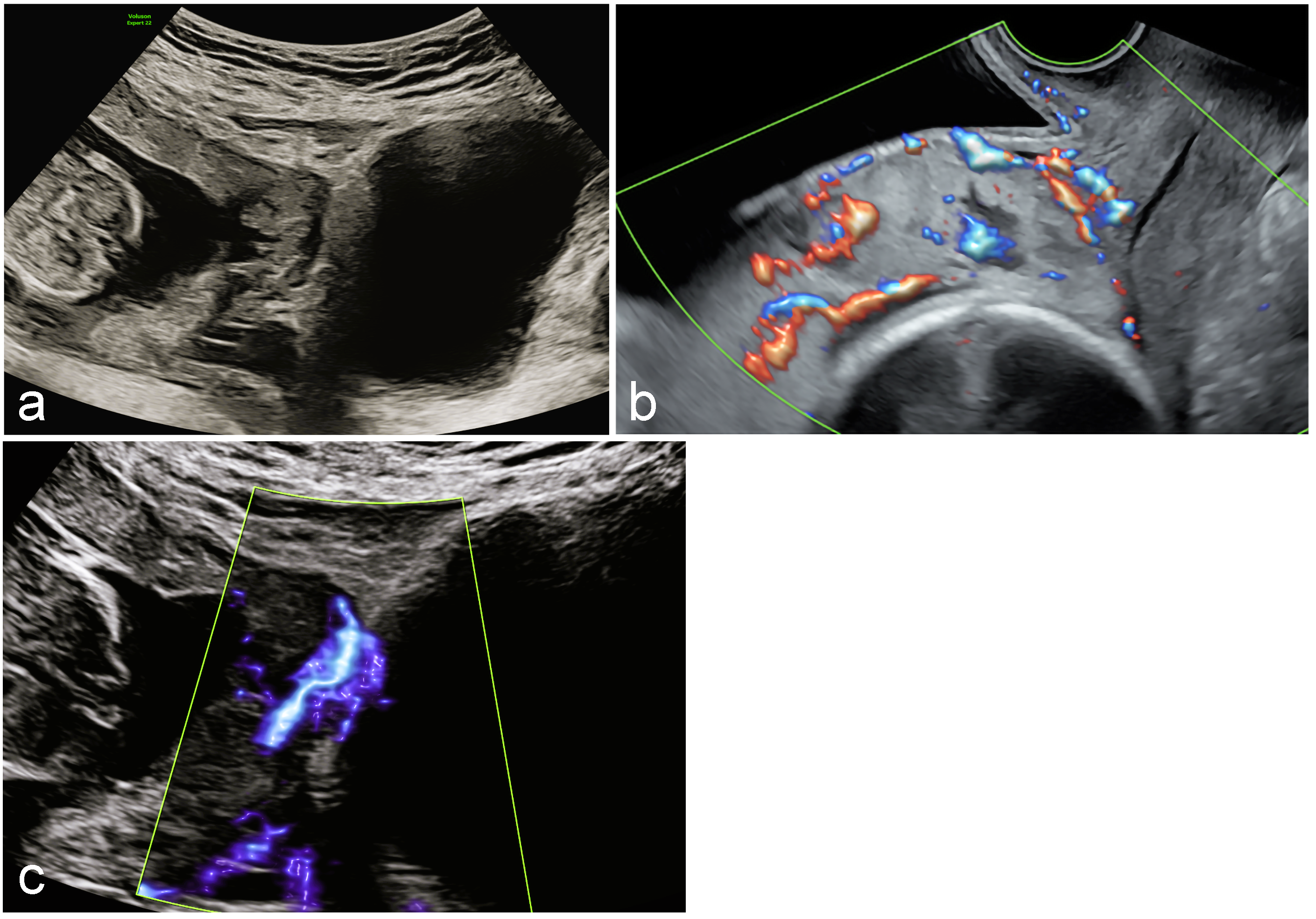

Supplement: SUPPLEMENTARY FIGURE 6 — Placenta percreta. Early diagnosis of placenta percreta at 14 weeks was made possible by the application of SlowflowHD. While the characteristic niche is visible in grayscale (a), the Doppler image (b) was suspicious for placenta accreta but did not show bladder invasion. SlowflowHD showed invasion to the maternal bladder (c). [file Image_6.tif]
