# Supplementary material for: Evolution of Doppler ultrasound in obstetric imaging: a slow(flow) step forward
Source: Front Med (Lausanne). 2026 Jul 17;13:1878231. doi: 10.3389/fmed.2026.1878231 (PMC13423865; doi:10.3389/fmed.2026.1878231)
Supplement: Supplementary file 7 [file Data_Sheet_1.pdf]

Supplemental Table 1: Added value of Slow*flow*HD shown in the Figures.

Figure title	Added value
**1a-c: Brain Vasculature**	Successfully demonstrates target vessels, as well as those not routinely visualized.
**Figure 1d-f: Precordial venous system**	Successfully demonstrates target vessels, as well as those not routinely visualized.
**Suppl Figure 1: Partial agenesis of the corpus callosum**	Successfully demonstrates target vessels, as well as those not routinely visualized. May show false continuation of target vessels.
**Suppl Figure 2: Cerebellar hyperechogenicity**	Successfully demonstrates subtle vascular findings, essential for diagnosis.
**Suppl Figure 3: Early pulmonary veins and the thy-box plane**	Successfully demonstrates target vessels at first trimester scanning.
**Suppl Figure 4: Coarctation of the aorta**	Successfully demonstrated vascular anatomy that was not visualized in HD-Doppler
**Suppl Figure 5: Tetralogy of Fallot**	Successfully demonstrated vascular anatomy that was not visualized in HD-Doppler
**Suppl Figure 6: Placenta percreta**	Successfully demonstrated vascular anatomy that was not visualized in HD-Doppler
